# Consumers' body image expressions: Reflection of a Snow White or an Evil Queen

**DOI:** 10.3389/fpsyg.2023.1097740

**Published:** 2023-03-17

**Authors:** Ralf Wagner, Swati Singh

**Affiliations:** ^1^DMCC-Direct Marketing Competence Center, University of Kassel, Kassel, Germany; ^2^Marketing Department, Vivekanand Education Society's Institute of Management Studies and Research, Mumbai, India

**Keywords:** minimal-self, fitness, fairy tale, body appreciation, social media, cosmetic surgery, mindful living

## Abstract

**Introduction:**

The aim of this paper is to explore how minimal-self impacts the body image, projecting it as a reflection of one's approach toward their health and mental well-being.

**Methods:**

The study takes qualitative data from two countries India and Germany and draws on a qualitative study of 20 individuals who are involved in some kind of physical activity for a long time. This paper examines the body image perspectives from *Grimms Brothers fairytale characters* showcasing fit and healthy perspectives on *Snow White* side and projected and superfluous perspectives on *Evil Queen* side. The study also provides a model deciphering the rationale for both the reflections.

**Results:**

The body image projection from Snow White perspectives (success & dedication, self-esteem, bodybuilding, and cosmetic surgery) relates to positive reflection of oneself with focus on fitness, discipline, and mental rejuvenation in life. Notably, Evil Queen perspectives (unrealistic makeover, dark side of social media, gain an edge over others, and mental benchmarking with fair skin) reveal these facets as motivators to equip their body as means of physical non-verbal communication assets.

**Conclusion:**

Analysis shows that there is no clear white or black view of health and fitness projection *via* body image but it's a gray line that gives wholesome fitness either a holistic mental peace or a competitive or success-oriented approach.

## Introduction

Body image is the reflection of the synchronization of overall fitness and mindful living (Marschin and Herbert, 2021; Balciuniene et al., 2022). Positive self-care, intertwined with rational self-talk, opens doors toward doubts and criticism about body weight and appearance. Individuals are prompted to adopt a well-balanced approach toward their health and wellness (Cash, 2008; Fernández-Bustos et al., 2019) or to go further in changing their bodies. A suited balance in turn provides support to positive construction of realistic life goals and self-projections.

Over the years, the body image construct has seen transience through varied cognitive components like perceptual experiences, self-awareness, and mindfulness (Vago and Silbersweig, 2012). Viotto et al. (2021, p. 2.) elucidated that the “body shape acts as a marker of distinction in status games.” Self-identification and societal projection have seen numerous benchmarks over decades (e.g., *Nike sneakers* and *Under Armor shirts* in the 1980s; *Lara Croft, Jennifer Lopez* in the 1990s; *Kim Kardashian* and *Kendell Jenner* in the 2000s). This epitome of contemporary lifestyle communication with the underpinning of love-your-body discourse embodiment of health with both *reel* and *real* models makes the trained body a status symbol that money cannot buy. In the contemporary context, body appreciation and its projection have become an explicative tool for self-esteem, proactive coping, and life satisfaction (Kuuru, 2022).

The minimal-self defines a scholarly concept of understanding oneself by the individuals that helps them to relate their being with others in the society (Musculus et al., 2021). Minimal-self of the people is not only confined to self-evolvement but is breaking the boundaries and imbibing the whole set of performance tools through body image presentation that can provide both leverage and appreciation in the society (Schilder, 2013). This dimension of performance, coupled with aesthetics, conveys concordance between appearance and health-giving impetus to competition and achieving excellence and success in both personal and professional spheres of life (Dagalp and Hartmann, 2022). This genre of performers are synthesizers of their physical appearance with mental satisfaction through the culmination of vocational training and professional skills that gives them an edge in the society (Webb et al., 2015).

This study investigates the modernized ideal body and mind fitness perceptions and consequent actions that can bring a change in the outlook toward life. It also deals with both positive and negative approaches of minimal-self that lead to a healthy or perfect body image based on an individual's perception of body image projection. In this vein, this study presents a general schematic model that identifies the cognitive resonance in respect of people's attitudes toward their physical and mental wellbeing. This study aims to cast an anthropological view on this facet of consumers' behavior that substantially absorbs lifetime, awareness, and cognition but also contributes to happiness and overall life satisfaction. The myth and magic underlying the globalized body culture are used to structure the relevant facts and overcome the limitations of reductionist utility-based behavioral modeling.

## Body image: A congruence of minimal-self

Physical appearance and body image play an important role in the pursuit of happiness and growth (Schilder, 2013). Body image is a complex phenomenon, with an individual's assessment of self-being a highly judgmental phenomenon seen through the lens of self-esteem, ideas, or feelings (Wykes and Gunter, 2004). Self-reflection and self-improvement are the important antecedents that cultivate both body appreciation and body improvement.

With the technological onslaught and omnipresent marketing approaches in all aspects of life, human beings are more accustomed to change than ever before. They do not falter at any point in life to grasp new things and enjoy evolving with them. This attitude is also driving their mental state to look at themselves with new perspectives and desire to do something different. Herein, the thought of entering unknown territories to explore one's minimal-self becomes both exciting and rejuvenating and heightens the sensory inputs for creating a body image that is different from age-old norms of gender attitude toward fitness and wellbeing. Currently, people see their ideal-self through new spheres of personality enhancement by overcoming normative societal barriers and breaking invisible boundaries to achieve and showcase their new avatar. Positive minimal-self is also leading to a paradigm shift in people's approach to handle work–life pressure and succeeding on both parameters (Tylka, 2011).

## Self-expression: Snow White vs. Evil Queen

Psychological literature talks about changing cognitive and/or behavioral paradigms for personal growth and stable mindset. Minimal-self is the key to decode the effects of body image on people's functioning or performance (Schilder, 2013). One's socio-cultural system prompts one to perform in a particular manner that provides both self-regulation and gratification in life, leading to personal development and growth.

The literature on body image talks about both positive and negative aspects of self (Cash and Fleming, 2002). This study roots these two aspects in the Grimm brothers' German fairytale *Snow White*, which deals with two characters with opposing personalities and outlooks (Grimm and Grimm, 1857, 1991, 2008). Faber (2022) explained the relevance of fairy tale characters in interpreting differences in human values, morals, and other psychological dimensions. She states that the fairy tales “mirror the worldviews and values not only of the storytelling individual, but of their time and culture” (p. 90). In the fairytale and all its adapted versions, Snow White is a character that is projected as self-regulated, righteous, and hardworking, whereas Evil Queen is successful and designed as ambitious, powerful, and self-centered. Though both share a common attribute of beauty, the reflection of beauty is psychologically very different for both of them, which encompasses the social life of individuals. When mapped to the psychological understanding of body image and its projection, this concept can prove to be either a *boon or bane* for one's existence.

## Self-consciousness

### Success and dedication: Professional and personal

A motivational state which is both positive and fulfilling brings vigor and dedication to one's work (Baumann and Kuhl, 2021). Commitment toward one's goal brings strong identification with self and prompts an individual to strive for both success and satisfaction (Bakker et al., 2008). In fact, a self-regulated positive self energizes and motivates people to achieve affiliation within society, enhance their self-worth, and attain success and power (Heckhausen and Heckhausen, 2008). The same principle applies to attaining fitness of both the mind and body. The appearance of the body becomes a manifestation of self-evaluation and a benchmark for achievement and satisfaction in both types of Snow White and Evil Queen.

A positive body image is an embodiment of a person's beliefs that are achieved by careful diet, exercise, and dedication toward one's body. Healthy beings, in their constant endeavor to look good and feel fit, indulge in a high sense of pride and their achievements of perfect body image spills over to their other endeavors both in social and professional life (Tu et al., 2022). A whole new paradigm shift has occurred where these individuals embody their positive body image to foster protective and health-promoting activities that can support resilience and the capability to face adverse conditions. Mahlo and Tiggemann (2016, p. 136) stated that “embodied individuals experience their body as a vital and integral part of their self-expression and power, and as central to their overall wellbeing.” Overall, the pathway of body embodiment reduces self-objectification, enhances mind–body integration that instigates a sense of physical empowerment, and enhances competence (Fernández-Bustos et al., 2019).

### Self-esteem: Fitness freak, proud, and health quotient

Self-love and contentment are important tools of self-esteem (Ryan, 1983; Sciangula and Morry, 2009). People indulge in physical activities that give both sensory and inert pleasures, bringing more confidence and a feeling of empowerment to themselves (Cash, 2008; Shang et al., 2021). The inclination toward body image predisposes a sense of meaningful involvement of minimal-self with overall lifestyle and brings acute consciousness among people who groom and worship their body to an extent whereby their fitness regime and healthy living become two sides of the same coin (Marschin and Herbert, 2021). In their pursuit of a fit body with a positive mindset, they imbibe a holistic and healthy lifestyle that enables them to love and respect their body (Tylka, 2011).

These individuals put much emphasis on their self-worth and appreciate and celebrate their physical selves and concomitantly develop respect for their mental and emotional wellbeing (Gillen, 2015). Their high self-esteem discerns them to consider themselves on a higher level, both on agentic and communal traits (Campbell et al., 2002). Their proactive and compassionate attitude toward their body and zeal toward fitness imbibes a proud feeling that encourages them to put effort into all the other areas of their wellbeing to have a healthier and robust lifestyle (Tylka and Wood-Barcalow, 2015). These fit individuals honor their bodies by becoming mindful of their incessant training, eating, and engagement in various physical activities.

Indubitably, body appreciation and self-esteem together form the base for wholesome satisfaction. Their high health quotient brings a holistically favorable perspective for their bodies prompting a strict fitness regime and bringing the feeling of empowerment that downplay any physical discrepancies they might have. Thus, these individuals with a positive mindset form a connection with their body and try not to fall into the social trap of body modification, nor do they try to adhere to cultural norms of attractiveness. Gillen (2015, p. 68) stated that “those with positive body image may be less interested in modifying their body to fit cultural norms of attractiveness, and consequently, may be less likely to harm the body in these efforts.”

### Bodybuilding: Self-identity and satisfaction

Body image is inextricably linked to body satisfaction, i.e., a person's own evaluation or self-worth (Clay et al., 2005; Ramos et al., 2019). In fact, people rejoice that their body supports them in their choice of healthy living and are thankful for getting a viable identity of their minimal-self (Monaghan, 2002; Ouyang et al., 2020). The synchronization of their fitness with their happiness gives them an outer radiance or glow that manifests positive energy to celebrate their imperfections rather than ponder over societal idols. Over the years, health and appearance have synchronized to become one entity marking them an integral part of *leisure* and *lifestyle* consumption. Correlating this, Maguire (2002 p. 449) elucidated “whether the concern is with the inner or outer body (or, more likely, both), fitness is represented as providing the answer: exercise makes you feel good and look good.” Self-improvement and self-identity are prominent cultivators of bodybuilding, which establish a congruous relationship between fitness and self-reward.

In fact, an engagement of the body with a fitness routine prompts the engagement of the mind leading to mindful eating and awareness of food and nutrition as nurturing agents rather than taste propellers (Viotto et al., 2021). Notably, body standards keep on changing with changing societal and wellness understanding. Previous decades showed different parameters of health-related behaviors. In the early 1990s, *jogging* and *running* were the health benchmarks; by the 2000s, going to the *gym* and *building muscles* (mostly by men) and being *thin* (mostly by women) became the new mantra for fitness. Interestingly, recent years have seen a visible transformation in the approach and acceptability of health standards with a more holistic paradigm body-related normative systems with yoga, meditation, physical activities, mindful living, and nutritional food along with organic and natural food consumption have become the buzzwords for leading a good healthy life (Lauche et al., 2017).

This also gave a riveting twist in the mindset, which is breaking the shackles of the society that brackets men and women in different realms of body image. Nowadays, there is no set rule for any gender and their behavior thereof. Notably, currently, men do not shy away from going for yoga, meditation, or Zumba dancing. Matching this, women are more inclined to go for adventurous sports, build muscles, and be “toned.” By doing so, they overcome the trap of being unhealthy and thin and focus on being fit and healthy.

### Cosmetic surgery: Wholesome-being

Modification and cosmetic enhancement of the body through a range of regimes and technologies lead to aesthetic healing (Nerini et al., 2019). People who are dissatisfied with their bodies look at them as their shortcomings and feel that in spite of their complete dedication and efforts, they are unable to achieve what they strive for because others are judging them on their physical attributes rather than their intellectual or personal level (Bonell et al., 2021). These individuals look at cosmetic surgeries as a way out of the unnecessary burden that they have to carry for their physical features of body image. They look at the *appearance orientation* as a brighter side of the surgery with which they can build more self-confidence and negate their disappointments of body dissatisfaction (Sun, 2018). Von Soest et al. (2009, p. 1239) also affirmed that “a cosmetic operation with an aesthetically successful result would lead to improvements in key psychological variables such as body image, self-esteem, and mental health.”

This study intersects two cultural angles to understand the acceptance or rejection of cosmetic surgeries. In India, harmony with nature and conservation are more important and are in sync with earlier ideology of nature's gift, whereas Germans are more open to accepting individual choices taking a liberal point of view, allowing for the changes that can bring forth recognition and success in life. Therefore, it can be said that cosmetic surgery that helps in correcting imperfections (nose job or feature enhancements) acts as an *appearance investme*nt and *morale booster* that increases many career opportunities and leads to personal enhancement by reducing discomfort and boosting the morale of a person (Wu et al., 2022). People tend to feel more beautiful, and their confidence gets boosted, which provides them with motivation and zeal to take on the challenges of the world. In this regard, cosmetic surgery brings higher feelings of acceptance and consequently prompts people to adopt the right style and desired looks that gives easy acceptability in the reference group and enhances one's status in society.

## Pressure of social recognition

### Unrealistic makeovers: Surgery, steroids, and extreme diets

In order to be fit, the body and mind need to synchronize at all levels. When there is a disconnect between both the mind and the body, dualism creeps in, with the mind acting as superior and active and the actual body being inferior and passive (Sobanko et al., 2018). This brings appearance-based sensitivity, which becomes a seedbed for risky ventures like body enhancement/modification surgeries, taking steroids, and going on extreme diets (Ricciardelli and White, 2011). Body contouring, liposuction, and Keto diets become part of this frenzy where achieving perfection, and global body image standards are the benchmarks for bringing joy in life, gaining self-confidence, attractiveness for life partners, and building social relations (Klassen et al., 2012).

Previous research distinguishes between individuals who are simply dissatisfied with their body or some facets of their perceived self and have to accept that it might take some effort to improve, e.g., doing more sports (Shang et al., 2021), and individuals who are going far beyond this. Societal benefits generally trigger the urge to go for makeovers, especially for people suffering from “body dysmorphic disorder” who feel anxious about their appearance and want to remove those flaws at any cost (Frederick et al., 2007). Altogether, unrealistic makeovers look like a good investment for the individuals who passionately monitor their appearance and are always at crossroads with the dilemma of accepting their body as it is or winning over the society with the right body image (Wu et al., 2022). When conformity to societal image projection wins, an obsession and continuous monitoring to get everything perfect takes root, pushing the individuals to go for these makeovers. However, winning over this dilemma and choosing the right path to fitness is not an easy road to travel, thus paving the way for shortcuts like surgeries, steroids, and fad diets. This study follows the modern view that the assignment is the groups of “being dissatisfied” (how much I am suffering and how stable).

### Dark side of social media

By creating an imaginary world with perfect bodies, beautiful, unmarked faces, and fascination for everything over-the-top, like difficult yoga postures, muscles, fair skin, and perfect hair (Thompson and Heinberg, 1999; Wykes and Gunter, 2004), the countermovement of body positivity was seeded. Nevertheless, social media images continuously create psychological pressure and make people susceptible to their bodies (Boursier et al., 2020). This encourages people to go for imaginary societal standards of having a perfect look (Pan et al., 2022), which are illusions created using filters and various photographic techniques that in real life are unachievable (Mingoia et al., 2017; Butkowski et al., 2019).

As the concerns for perfect body images are increasing, it can set the ball rolling in a completely different direction from fitness and health and cause adverse psychological effects like bulimia behaviors with overvaluation of thinness and weight (Heider et al., 2015), social physique anxiety (Stojcic et al., 2020), body dissatisfaction, and fad diets (Krane et al., 2001). The negative approach toward one's body image makes people vulnerable and prompts people to undergo body modifications, Lasik treatments, and other surgical procedures (Grogan, 2016). Studies have found that this approach rarely provides confidence and positivity for long as they are promoted from a negative mindset (Di Gesto et al., 2022). However, if surgical and other body improvements are done with a positive aspect of *minimal-self* involving being fit and healthy, it gives a long-term sustainable approach to achieving *ideal-self*. Contemporary research on masculine femininity mainly focuses on social media by appearance and the effects on others (e.g., Ferguson et al., 2020) but does not cover the real-world experiences of leading class performers. Interestingly, neither their individual target metrics and their plans nor their daily routines for achieving their aims have been considered so far.

### Gain an edge over others

Digitalization has brought in a new era of competitiveness with virtual reference groups through Facebook, Instagram, and YouTube connections (Manyika et al., 2016). People use these para-social relationships to gain an edge over others by posting the perfect videos and photos, which can bring high social recognition and help establish their mark in the society (Kim, 2021). This can be related to the Evil Queen's side with over-the-top image projection and societal enhancement. In real life, similar results can be achieved by lifting heavier weights, gaining more muscles, or slimming down faster for the perfect beach body (Viotto et al., 2021).

Researchers have stated that life's outcomes and perceptions of an individual are directly related to physical attractiveness. Attractive people are judged as being more competent than their less attractive peers. In the social circle, attractive people with good physiques stand out and are considered smart and capable (Tu et al., 2022). Regardless of being more intelligent, capable, overweight, or less attractive, individuals get stigmatized as stupid and sloppy. Society shuns them by labeling them “lazy cows” who are not to be bothered with (Puhl et al., 2008). This pressure leads to anger and frustration, which might lead these individuals toward unrealistic makeovers to win the rat race.

### Mental benchmarking with fair/tanned skin

Society as a whole makes some unrealistic and irrelevant benchmarks of beauty and looks that individual's try to cater to in order to get higher recognition and acceptability (Charles and McLean, 2017). These societal benchmarks consider the body as a commodity and often ride on totally baseless constructs like looks and the color of the skin. The fascination with aesthetics is perpetuated by cultural and nonsensical beliefs that make *colorism* an important aspect of acceptance.

This obsession and inclination for fair skin reify the agony of dark-colored people who are stigmatized and rejected by many Asian-American societies (Mucherah and Frazier, 2013). Colorism prompts discrimination against dark-skinned people regarding their professional growth, their position in society, and marriage prospects (Hunter, 2007). This undesired fascination with fair skin is also reaping huge profits for big corporations selling fairness creams or for doctors performing skin-lightening procedures (Hunter, 2011). The completely irrational approach to *lighten, brighten, or whiten* the skin does not adhere to practical protocols; rather, it thrives on social rewards and gains from societal alignment and subsequent acceptance.

Interestingly, the opposite is in vogue in Europe. Contrary to brown-toned people who obsess over fair skin, white-skinned people obsess over tanning (natural or artificial) to achieve and leverage societal acceptance/gains (Rondilla and Spickard, 2007). The growing fascination with tanned and darker looks has increased the attraction for *sunbeds* throughout Europe which can give an attractive look and improve self-presentation (Suppa et al., 2019). In fact, in Germany, the dominating projection of healthiness and actively enjoying life comes with being tanned. Furthermore, Diehl et al. (2021) elaborated that the use of sunbeds is more prominent among employed individuals as they are in the competitive market and do not want to lag on any parameter of competition, talent, or aesthetic representation. They also concluded that young women are more eager toward artificial tanning to gain leverage over others with their appearance and self-confidence.

## Methodology and framework

This study uses a qualitative interview method to understand the perspectives on body image. The questionnaire was designed to garner understanding and perception of people regarding their body image projection in relation to the fictional characters created by the Grimm brothers in the story “Snow White” (Grimm and Grimm, 1991). The qualitative interviews give fresh and novel perspectives to the scholarly world on understanding the personality and behavior thereof through the lens of these fictional characters. Mapping the minimal-self of the individuals with their body image reflection, this study provides a strong methodological fit to decode people's end goal when they indulge in physical activities.

The study is conducted in India and Germany with the basic understanding that it would be similar for people across the world who, with their cultural, economic, or societal differences, have similar apprehensions and problems. The skeletons in people's closets and their purview of dealing with those issues are the same across the nations. Participants were told that the purpose of the study was to learn about their attitudes and behaviors related to body image reflection and satisfaction.

### Data gathering and evaluation

From January to March 2022, 13 Indians and seven Germans participated in semi-structured interviews. Following the procedure described by Ritchie et al. (2009), they were non-randomly selected in a purposive manner. They were sampled from various sectors and different educational backgrounds, ranging from students to young professionals to middle-aged people (Table 1). They were contacted directly *via* email or WhatsApp. Subsequent to their consent to participate in this study, they were assigned to a group actively doing sports training activities on a regular basis or not. Thus, researchers got informed from both perspectives: active “self-optimizers” and passive “self-acepters” to cover the whole range of motives and individual judgments. To accurately contextualize the test subjects' answers, it is important to understand the situations in the two countries. Germany and India were chosen as examples because these cultures provide a sound contrast (Timokhina et al., 2018) that enables us to distinguish between culture-bound motivations and appraisals and the universal ones that are shared by fitness enthusiasts across cultural contexts. Notably, the majority of related research is building upon evidence gathered in North America, and the research context at hand promises to widen the scholarly view. Recruiting stopped when the last two interviews did not add substantially new insights. The Indian respondents were heterogeneous in their motivations in contrast to the Germans resulting in an imbalanced sample.

**Table 1:** Sample description.

**Interview**	**Gender**	**Age**	**Fitness Rating for themselves**	**Qualification**	**Occupation**	**Country**
1	Male	44	9	MBA	Banker	India
2	Male	38	7	MBA	Businessman	India
3	Male	51	7	Doctorate	Professor	India
4	Male	25	7	Masters in software engineering	Salaried Professional	India
5	Female	47	6	Masters in chemical engineering	Homemaker	India
6	Female	36	6	MBA	Salaried professional	India
7	Male	50	6	Doctorate	Consultant	India
8	Female	27	7	MBA	Digital marketer	India
9	Male	29	6	MBA	Assistant professor	India
10	Male	45	7	LLB	Lawyer	India
11	Male	43	8	Masters in civil engineering	Business	India
12	Male	44	6	Masters in mechanical engineering	Banker	India
13	Female	29	6	MBA	Data analyst	Germany
14	Male	27	10	Masters in yoga science and naturopathy	Yoga instructor	India
15	Female	39	7	Doctorate	Professor	Germany
16	Female	30	6	Masters in business studies	Research associate	Germany
17	Female	31	9	Masters in social science	Research assistant	Germany
18	Female	28	7	Masters in physical education	Fitness trainer	Germany
19	Male	45	8	Masters in business studies	Research associate	Germany
20	Male	29	8	Masters in psychology	Research assistant	Germany

**Figure 1:**
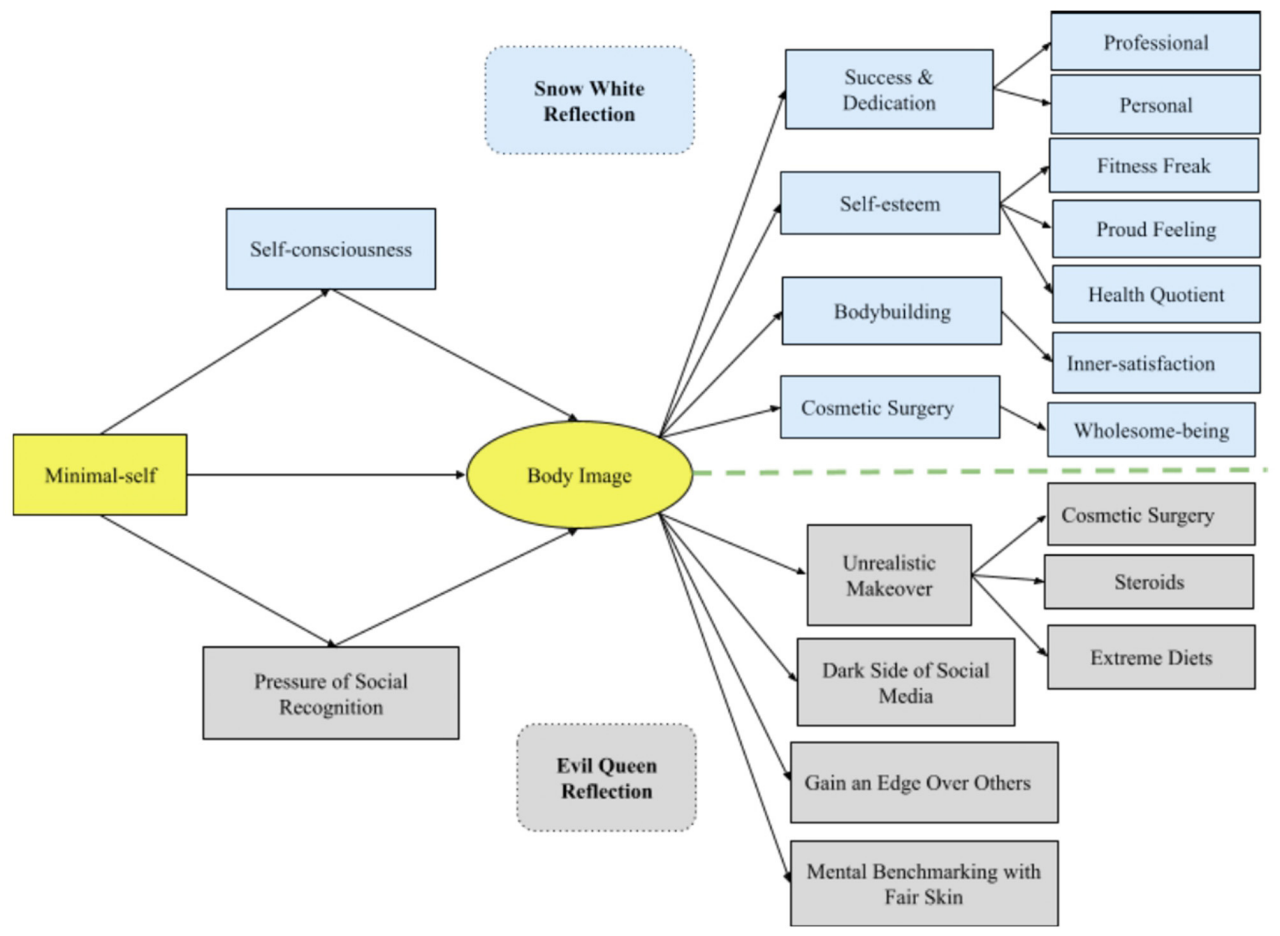
Body image expression.

All answers from the respondents were saved using Google forms. In the first step, the answers were checked for consistency and completeness. The responses were assigned to the concepts depicted as consequences of the body image in Figure 1. According to the answers to our questionnaire (Supplementary Appendix 1), some, but not all, of the concepts subcategorized, as depicted in the very right side of Figure 1, e.g., Self-esteem is broken down into “Fitness Freak,” “Proud feeling,” and “Health Quotient.” All analysis was done using a Google spreadsheet with independent coders and one “validator” checking for inconsistencies. The use of a spreadsheet instead of specialized software, such as MaxQDA, and NVivo, is motivated by the fact that the research aim is to drill down to the motivations and judgments underlying respondents' actual routines rather than defining second or higher order categories.

## Results

### Snow White perception

As predicted by the Snow White reflection in the conceptual model, the self-consciousness of all respondents relates to a positive reflection. Physical activities and a history of going to the gym contribute to stabilizing the self-concept and pride: Respondent #12 stated that “*I walk daily. It's been many years since I've been doing it and it is now an essential part of daily activity*” and #15 argued that “*I have been doing this for the past 5 years and I love it now*.”

Notably, none of the respondents reflected negatively on his/her history of fitness activities or gym memberships (e.g., not achieving the expected results, paying membership fees without practicing, etc.). On the contrary, they suggest setting their level of activities as a social norm for others: Respondent #3 said that “*I believe that irrespective of one's profession and age, fitness should be part of life to gain an edge in all aspects of life.”*

After getting used to the gym or substitute activities, the respondents integrated them into their routine, achieving physical and mental rejuvenation. Respondent #1 revealed that “*For fitness I have been going to gym regularly and when I can't go, I take a walk or do yoga. I rarely miss,”* #2 conveyed that “*Going to gym makes me both physically and mentally fit*,” #13 alleged that “*I go to the gym 4–5 times a week for multiple reasons like staying fit, getting in shape, eating without feeling guilty and maintaining my mental balance,”* and #20 said that “*I go to the gym to stay physically healthy and to achieve balance at my job and sports*.”

Like “appetite comes with eating,” the fun of activities in the gym comes with practicing and getting used to it. Adrenaline rush for the body and social contacts/belonging, in addition to the inner satisfaction of having completed action for yourself (self-rewarding) and for the mind. Notably, in the analysis, no differences between the set of German and Indian respondents were observed with respect to the capitalization of Snow White activities. This result is remarkable because the current changes of emphasizing sports and leisure time activities over hard work and professional success have been manifested (Csikszentmihalhi, 2020) for the German cultural context, whereas the emerging Indian society provides an embedding culture naturally emphasizing economic activities over self-development (Pradhan et al., 2021).

### Success and dedication

The results of fitness training are perceived as a success by themselves rather than a means to succeed in private or professional lives. However, the attitude to physical achievements goes along (might be “generalized to” or “is taken” from) to manifest accolades in private and professional life.

Respondent #1 stated that “*Without discipline one cannot achieve the set goals*.” Respondent #3 said that “*Every successful event requires discipline and the same goes for fitness. There is no shortcut for hard work. Dedication certainly brings fruit to one in the form of success.” [SIC]* These statements indicate another relevant facet. Practicing fitness is not always attributed to fun, excitement, or relaxation but also to discipline to achieve a certain goal. Sports activities serve various educational purposes in kindergartens and schools, e.g., learning about fairness, team building, and inclusion. This vein is reflected in the mindset of some German respondents, e.g., respondent #13 argued that “*...it can surely help to shape your personality and make you feel more competitive and enhance self-esteem.”* Meeting this view #16 said that “*Yes, a good and fit body is the source of health and good performance in social and professional life.”*

Complementing the goal of developing one's own personality, the effects of fitness activities are appreciated by the respondents, e.g., respondent #12 answered that “*A well-maintained body will always help in creating the initial good impact” and* #15 confirms the view that “*It keeps one feeling light and helps one to be active with daily life chores.”*

The relationship between fitness and discipline is twofold. First, physical fitness is an indication of a disciplined personality in the vein of biosemiotics. Second, discipline is confirmed to be key to achieving and maintaining this non-verbal communication asset. Respondent #10 claimed that “*I wholeheartedly agree that one can't miss the routine. It is what keeps one dedicated to their goals,”* #2 own consolidated experiences “*Yes, constant work-outs without larger breaks are key*,” and #14 confirmed that “*Having a routine helps with consistency.”* These respondents support the claim introduced in the above literature review that self-surveillance for physical fitness enhances self-control and leads to monitoring and channelizing of inner strength to continue in the right direction.

However, not all respondents agreed to generalize this view: Respondent #16 said that “*I think sports need to be fun and you do it because you love it and not because of good mental discipline. I think the key is-fun.”* Consequently, the interpretation of fitness training results might diverge between the extremes of being more disciplined than others (Evil Queen) or looking backward to a history of more training enjoyment (Snow White).

Considering the cultural framing of the respondents, the analysis indicates that Indian respondents are more likely to consider their activities as a means to another end, e.g., demonstrating self-discipline, whereas the Germans are far more likely to explicate fun and enjoyment.

### Self-esteem

The respondents' perceptions of self-esteem by means of fitness activities turn out to be quite asymmetric. Only respondent #1 admitted that “*I feel guilty when I miss my workout*.” All other respondents had quite a positive perception of fitness training for self-esteem, e.g., respondent #7 claimed that “*If I don't exercise it makes the day dull whereas exercising gives me positive energy which helps throughout the day”* and #9 argued that “*I feel proud after looking at the results I have achieved after engaging in physical activities.”* Respondents supported the synchronization of physical fitness workouts with their perceived self; e.g., respondent #10 stated that “*Physical activity can surely help to shape personality and make one more competitive and enhance self-esteem.”*

Notably, the analysis did not indicate substantial divergences in the perceived contributions to the respondents' self-esteem between the Germans and the Indians.

### Bodybuilding

The self-rewarding behavior turns out to be a relevant driver of the fitness-related lifestyle. Respondent #4 claimed that “*One should always take out some time in a day for self* ” and #17 argued that “*One must take time for oneself and do what they feel like doing*.” The latter statement is hinting to a fact latently read between the lines of all interviews: the rejection of perceiving oneself to be passive. Almost all the respondents reflected that they definitely feel proud and empowered when they engage in some kind of physical activity; e.g., respondent #17 said that “*Yes, especially when performance improves, and you can achieve sporting succes*s.” Only one respondent #7 stated that “*I am not exactly sure that feeling of pride is related to physical fitness. It is just a routine that some follow rigorously, and others are not much bothered.”*

Notably, the respondents confirmed their appraisal of the positive mental changes resulting from their fitness activities. Respondent #2 said that “*I* a*gree that a fit body leads to enhanced confidence and overall happiness and satisfaction”* and #18 claimed that “*Sport and a corresponding body are not the most important thing when it comes to inner satisfaction and happiness but contributes with a part.”*

Physical and mental processes are synchronized to become one entity contributing, and integral component of the minimal-self that has an impact on the lifestyle, particularly on health-related consumption, e.g., respondent #2 informed that “*When you feel your muscles after training you would try not to eat irresponsibly”* and #5 said that “*physical shortcomings can be overcome by working on self and by compensating through excellence in some other area*.” However, not all respondents confirm this direct link; e.g., respondent #17 said that “*These two things have only a limited connection with each other. Mindful eating is fundamentally an important aspect, but I see it as separate from my sporting activities”* and #18 said that “*I make sure to eat a little more on sports days than on other days. But I also pay attention to healthy eating independent of sports.”*

Consolidating, physical activities lead to inner satisfaction and for some, but not all, respondents, this is triggering mindful consumption in related domains such as nutrition. The contribution to respondents' satisfaction and happiness emerges from the mental alignment and appears to be invariant to the embedding cultural context.

### Cosmetic surgery

The Indian respondents argued that imbibing traditional methods like yoga can bring positive changes in the body. They are aggressive in their approach toward natural looks. Respondent #6 said that “*Cosmetic surgery is completely bizarre. As everyone is UNIQUE in their own way, then why rectify it and become clones of one anothe*r.” However, some argued that if it gives satisfaction then people can go for it. Notably, most respondents took a quite liberal viewpoint, e.g., respondent #13 argued that “*I would not do it nor recommend it. Yet, I try not to judge people who feel the need to do it (to a certain extent I find it okay)”* and #17 said that “*It depends on the shortcomings and the impact on one's own wellbeing. In principle, I reject such operations for myself, but if people need these operations due to mental health, then they should be performed.”*

The respondents tolerating cosmetic surgeries for others, but not for themselves, might keep their mental door open to accept these “biosemiotics technologies” (Askegaard, 2021) for their future life when conservative procedures such as yoga and fitness training do not compensate for the body's deterioration anymore because of aging. Although German society is a rapidly aging society, whereas Indian society takes advantage of having one of the youngest populations in the world, the analysis did not reveal substantial divergences in the appraisal/rejection of cosmetic surgeries at the respondent's individual level. However, biosemiotics technologies are still in an infant state. Therefore, cultural framing effects might rise in very near future (Wheeler, 2016; Arnould et al., 2021).

## Evil Queen perception

### Unrealistic makeovers

Although these negative facets are mostly judged as negative, the respondents would accept them, if reasonably justified, e.g., respondent #6 claimed that “*it may be acceptable for people in showbiz as their earnings depend on their looks but for normal people, it would be like living a fake life to impress strangers” and* #19 said that “*Maybe. At least if it is done right, accompanied by experts/doctors/etc.”* Interestingly, respondent #4 fitted the unrealistic makeovers to manipulate the mental state in the direction of the minimal-self: “*Yes sometimes if it gives you the success that you desire*” and #20 emphasized the intrinsic motivation “*I completely reject these measures if their only intention is to please others.”*

Following this, unrealistic makeovers come from the internal feeling that one is inferior, and one strives to meet acceptable standards of society. In this vein, they struggle to accept their body identity and try to manifest themselves as another being. This psychological battle is seen in both countries, paving the way for various body procedures.

### Dark side of social media

The respondents were smart in coping with the external influences of social media. Respondent #7 framed this influence in a positive way “*Social media is helping in betterment of body image but is also making people uncomfortable in their own body. I enjoy fitness more than running for unachievable goals*” and #16 said that “*It helps me to get my motivation back if I lose it. […] I'm more into food inspiration on Instagram.”* Respondent #2 stated that “*social media has a great influence in inspiring people but we need to filter the tips and hints posted on them.”*

However, the negative sides have been acknowledged by some respondents as well, but in a positive framing: respondent #12 said that “*It shows too much perfection, which people try to reach. It can be a good motivator but sometimes brings undue stress”* and #18 said that “*Yes, it is positive as motivation but negative as pressure*.”

Consolidating the respondents were aware of the social pressure. However, by either rejecting the norm or, most interestingly, framing the pressure as a motivational component, they felt protected. They did not perceive themselves to be a “victim” of the pressure. Notably, the Indian respondents emphasized the framing factors (food, beautiful scenery for their selfies) meeting recent trends in Indian middle-class priorities (Singh et al., 2020; Singh and Wagner, 2022).

### Gain an edge over others

Similar to the social media influence, the respondents did not deny the Evil Queen's ambition to gain an edge over others but framed this behavior in a positive vein again. Respondent #11 reported that “*Having a good physique always makes a difference in life*.” Respondent #8 said that “*Good physique gives more confidence and rather than gaining edge over others, it gives an inert satisfaction if both inner and outer body is fit”* and #14 said that “*Training and consistency are key to any achievement. Those who work hard, most likely, have an edge over the rest.”* Interestingly, physical fitness was related to mental and cognitive abilities. Respondent # 12 said that “*A perfect body is not enough for gaining advantage over people. A brilliant brain too is required.”*

In conclusion, Indians are clearly more influenced by societal and lifestyle projections. This is attributed to the fact that over the past three decades, the Indian demographic profile has changed, and they are more likely to get societal approval for their routines (Singh and Wagner, 2019). With increasing disposable income, they are adapting new paradigms of consumption (Deodhar et al., 2019) and are more open to new ways of living (Singh and Srivastava, 2018). Body image perfectly fits this new norm that brings personal sensibility in alignment and synchronization with societal expectations. Contrastingly, German society recently grasped the modern trend of body acceptance (Rosenbaum et al., 2021; Schlüter et al., 2021), tuning down the pressure on individuals to strive for unrealistic makeovers.

### Mental benchmarking with fair skin

A remarkable result is that the Indian respondents (exception is #15), who were all enthusiastic about their fitness and their achievements by means of their bodies and their mental advancements, all played down the relevance of fair skin, e.g., respondent #1 said that “*It's not important in today's era. No one should go for a surgery just to achieve skin color*,” #5 said that “*Fair skin is a fad which is nowadays less relevant. A FIT BODY is a BEAUTIFUL BODY*,” and #2 claimed that “*Color and texture of the skin should be of minimal importance. Physical fitness should be given due priority.”* Notably, these two exemplary answers differ by means of expressing the perceived minimal-self (#1 and #5) and normative view (#2).

Similarly, the European respondents played down the relevance of being tanned: respondent #20 said that “*A tanned skin is not as important as physical appearance and does not justify cosmetic procedures.”*

Interestingly Indian respondents have clearly showcased that they are evolving in terms of judging people by their skin color. Though Indian society is still riddled with the old-age system of skin color prejudice, the new-age people are trying to overcome this biased approach. They realize the importance of healthy and fit living. The German respondents still feel the pressure to be tanned, however carefully aligned to the recent societal movement of tuning down the relevance of tanned skin.

## Discussion, conclusion, and future research

In the vein of Askegaard (2021, p. 99), this study aims to contribute to a “physiologically and anthropologically informed understanding” of consumers' fitness routines and related practices. Although the research design explicitly addressed the Evil Queen facts, the first remarkable result of this study is that none of the respondents reflected negatively on his/her history of fitness activities or gym memberships (e.g., not achieving the expected results, and paying membership fees without practicing). Their perception appears to be phenomenal because discipline following a workout plan and specialized diet regime was agreed upon by respondents across the two cultures under consideration. Meeting this assessment, the resulting body was considered to be a non-verbal communication asset that justified all the burdens of achieving and maintaining it. The respondents, particularly the Indians, agreed that their trained body supported them in advancing their professional and private lives. Results indicate a double jeopardy effect, where the fitness training activity by itself was already leading to a mental rejuvenation, in addition to being equipped with their body by means of the physical non-verbal communication asses. Adding up, European respondents reported a positive effect by improving their self-confidence. Intersecting the two cultural angles of explaining the acceptance or rejection of purposive body modification reflected in the Evil Queen metaphor, the empirical analysis revealed that respondents from both cultural backgrounds admitted to being influenced by these components. Notably, with the exception of the unrealistic makeovers, the perceived pressure turns out to decrease in both cultures. Similarly, respondents' reflections on the Snow White metaphor revealed substantially more similarities than divergences that can be attributed to their cultural framings.

Consolidating, physical activities lead to inner satisfaction and for some, but not all, respondents, this is triggering mindful consumption in related domains such as nutrition. This result supports the claim of Askegaard (2021) that the relationship between health-related experiences and nutrition class for further research combines both psychological and anthropological perspectives. Particularly, the Evil Queen components are promising candidates to challenge the nutrition behavior (Gu and Ming, 2021).

The Snow White components in the results indicated support in developing one's own personality; however, the goal in this dimension remained unclear in this study. These results suggest one avenue for further research: sports activities' contributions to learning about team building, fairness, and inclusion, along with building self-confidence and coping with failures and frustration, is well elaborated in the contest of psychology in kindergartens and schools, but less research addresses these effects for elderly people and the authors are not aware of any study clarifying how the consumer set their goals complementing their physical fitness goals.

Interestingly, the deconstruction of the body as an object through social media does not always bear negative fruits. It also brings good and positive orientation among people to go for healthy living and mindful eating. Many fitness trainers, yoga gurus, and nutritionists have a cult following, which brings acute changes in people's life and way of living. The people are not only motivated by following these social media influencers but get reassurance that the path and lifestyle of healthy living they have chosen for themselves is correct and gives an extra push to keep going in the right direction.

O'Donnell et al. (2016) elaborated on women's understanding of the importance of appearance, especially in their workplace. Attractive women are socially more accepted and are considered more competent and intelligent than their less attractive counterparts. Our results confirm this observation, but going beyond, this rationale appears to be valid for the men as well. Notably, this rationale turned out to be a highly relevant element of consumers' appraisals in both cultures.

Women mostly get the maximum ambush based on skin color discrimination making them either less confident or pushing them toward an unrequited quest for getting fair skin through surgical procedures. This horde of *impression management* has become the key to unlocking success and gaining self-confidence (Viotto et al., 2021). The respondents played down the relevance of skin color by emphasizing the relevance of their physical but not their mental fitness. Another interesting result is the tendency to accept cosmetic surgeries if justified with a sound reason. Further research could clarify whether fitness enthusiasts are more liberal, or a hidden motivation is to keep the door open to their own procedures in their future fitness careers.

Similar to what marketers do by focusing more on packaging to make the goods more eye-catching and interesting, people in their life are using various tactics to stand out from the crowd and please everyone. A remarkable result of the study is that the respondents from both cultures feel protected from becoming a “victim” of social pressure. A smart metal strategy is their framing of social pressure as motivation. However, this might become a first step into a difficult scenario of over-motivation and get closer to the full range of Evil Queen components considered in this study. An enlightened view on this given by respondent *#16:*

“*In the end: Happiness is a choice (not a perfect body).”*

## Data availability statement

The raw data supporting the conclusions of this article will be made available by the authors, without undue reservation.

## Ethics statement

Ethical review and approval was not required for the study on human participants in accordance with the local legislation and institutional requirements. The patients/participants provided their written informed consent to participate in this study. Written informed consent was obtained from the individual(s) for the publication of any potentially identifiable images or data included in this article.

## Author contributions

Both authors listed have made a substantial, direct, and intellectual contribution to the work and approved it for publication.
