# Supplementary material for: Consumers' body image expressions: Reflection of a Snow White or an Evil Queen
Source: Front Psychol. 2023 Mar 17;14:1097740. doi: 10.3389/fpsyg.2023.1097740 (PMC10064093; doi:10.3389/fpsyg.2023.1097740)
Supplement: Supplementary file 1 [file Table_1.docx]

**Appendix I |** Body Image Projection Scale

| **Dimensions** | **Factors** | **Items** |
| --- | --- | --- |
| **Self-**  **consciousness** | Why do you go for gym/yoga/walk/exercise? How often do you do it? How long have you been doing this? | |
|  | ***Success and dedication*** | Do you think that a good and a fit body can help you in having an edge in both your social and professional life?  Do you agree that discipline is the key to fitness success?  Do you feel guilty or become restless if you do not take time out for yourself and your fitness activity? |
|  | ***Self-esteem*** | Do you feel proud that you engage in physical activities?  Making time for going to the gym is a self-reward after a day of hard work.  Does training in the gym/fitness make you more mindful of your eating and overall well-being? |
|  | ***Bodybuilding*** | Do you think that a fitness regime/building a nice body brings inner-satisfaction and happiness to self? |
|  | ***Cosmetic surgery*** | Do you believe that a person should go for cosmetic surgery to correct any physical shortcomings? |
| **Pressure of social recognition** | ***Unrealistic makeovers*** | Is going for steroids/extreme diets/cosmetic surgeries and other makeovers acceptable if they help in success or social recognition? |
|  | ***Dark side of social media*** | Do you believe social media is inspiring people to go for body sculpting and perfect physique?  Do you enjoy fitness tips and hints posted on social media? Do you follow any fitness influencer?  Is social media pushing people to go for body sculpting and perfect physique? |
|  | ***Gain an edge over others*** | Does a perfect body help in beating competition and gaining advantage over other people?  Do you expect differences of this effect in your private life and your professional life? |
|  | ***Mental benchmarking with fair skin*** | Is fair skin more important than physical appearance? Does cosmetic procedure justify getting light-skinned? |
|  | On a scale from one to ten: How satisfied are you with your body/current fitness level? What needs to be improved? | |
|  |  | |
